# The Earth Microbiome Project: The Meeting Report for the 1st International Earth Microbiome Project Conference, Shenzhen, China, June 13th-15th 2011

**DOI:** 10.4056/sigs.2134923

**Published:** 2011-11-22

**Authors:** Jack A. Gilbert, Mark Bailey, Dawn Field, Noah Fierer, Jed A. Fuhrman, Bin Hu, Janet Jansson, Rob Knight, George A. Kowalchuk, Nikos C. Kyrpides, Folker Meyer, Rick Stevens

**Affiliations:** 1 Argonne National Laboratory, Argonne, IL, USA; 2 Department of Ecology and Evolution, University of Chicago, Chicago, IL, USA; 3 Centre for Ecology & Hydrology, Natural Environment Research Council, Crowmarsh Gifford, Wallingford, Oxon, UK; 4 Dept. of Ecology and Evolutionary Biology, University of Colorado, Boulder, CO USA; 5 Cooperative Institute for Research in Environmental Sciences, University of Colorado, Boulder, CO USA; 6 Dept. of Biological Sciences, University of Southern California, Los Angeles CA USA; 7 Beijing Genomics Institute at Shenzhen, Guangdong, China; 8 Lawrence Berkeley National Laboratory, Earth Sciences Division Berkeley, CA USA; 9 Howard Hughes Medical Institute and Department of Chemistry & Biochemistry, University of Colorado at Boulder, Boulder, USA; 10 Department of Microbial Ecology, Netherlands Institute of Ecology (NIOO-KNAW), Wageningen, The Netherlands; 11 Department of Ecological Science, VU University Amsterdam, Amsterdam, The Netherlands; 12 DOE Joint Genome Institute, Walnut Creek, CA, USA; 13 Computation Institute, University of Chicago, Chicago, IL USA

## Abstract

This report details the outcome of the 1st International Earth Microbiome Project Conference. The 2-day conference was held at the Kingkey Palace Hotel, Shenzhen, China, on the 14th-15th June 2011, and was hosted by BGI (formally the Beijing Genomics Institute). The conference was arranged as a formal launch for the Earth Microbiome Project, to highlight some of the exciting research projects, results of the preliminary pilot studies, and to provide a discussion forum for the types of technology and experimental approaches that will come to define the standard operating procedures of this project.

## Introduction

The Earth Microbiome Project [1-3] is an ambitious endeavor that aims to generate the largest repository of comparable environmental sequence data yet attempted. The EMP is driven from a fundamental need to understand life on Earth and its interactions with the environment. To achieve this, it has become clear that we need a deep exploration of the microbiome of Earth through systematic characterization of the microbial communities and their diversity across the planet. The need is fueled by scientific and economic justifications for a large-scale and rapid assessment of global microbial biodiversity. The technical challenges associated with sample acquisition, data generation and and data analysis are essentially issues of scale and can only be resolved with sufficient support from the community and the funding agencies.

The benefits of generating a comprehensive planet wide survey of comparable data are many fold, including an unprecedented knowledge resource that will allow fundamental advances in the study of microbial biodiversity, biogeography, ecology, global protein and gene diversity, evolution and community dynamics. Advances in sequencing technology, coupled with advances in computing and data analysis and the rise in massively-parallel researcher communication networks (social networking science), makes it possible to now consider a distributed and scalable approach to the problem of sample collection, processing, sequencing and analysis for hundreds of thousands of environmental locations.

The 1st International EMP Conference was designed to showcase the rationale, the tools, and design of the EMP, highlighting the technical challenges, and the potential. The EMP defines a suite of standard protocols and procedures for the processing and analysis of thousands of samples from disparate environments and locations. While the ‘no-size-fits-all’ paradigm is a fundamental problem for any global survey, the benefits of generating such survey outweigh these complications. Generating an integrated understanding of the role of microbes in the ecosystem turnover of each system on earth, and exploring the complexity of interaction between each system will help to define and build a new model of Earth’s biodiversity, which will help to define and refine our capability to manage the resources of this planet.

The main goal of the EMP is a  systematic characterization of microbial life on Earth, which is exceptionally challenging and is comparable, if not exceeding, the challenge faced by astrophysicists and astronomers in exploring the universe. There are approximately 5 x 10^30^ microbial cells on Earth [4,5] which is a billion times the number of stars in the known universe [6], and their genetic complexity is exceptional and is both cause and effect of their ubiquity in every niche on Earth. Yet, no ocean is bottomless, and the number and type of functional adaptations to environmental conditions must be finite even if in flux. However, while it is vital that we understand the players and plays associated with the microbial world, this census is only a small aspect of the EMP. One of the main goals is to generate a suite of microbial community models that enable us to predict, for example, the changes in metabolite turnover in diverse environmental systems over different spatial and temporal scales to help us better manage our environment plan and mitigate future changes in the environment, e.g. climate change.

The 1st International EMP Conference was an open meeting with over 100 attendees. There were 8 invited guest speakers, including Rick Stevens (Argonne National Laboratory and University of Chicago) who gave the keynote on the morning of the first day. In addition, we had 22 offered talks with presenters from a range of nations (China, USA, Germany, France, New Zealand, Australia, and Spain). The meeting was loosely divided into two themes. The theme for Day 1 was microbial ecology, which focused on why we need the EMP, tools and models for the EMP, some preliminary data from the pilot study, and a number of exciting case studies from EMP collaborators. The theme for Day 2 was chiefly dedicated to standards and bioinformatic techniques, which included novel data analysis tools, standard data acquisition, and some considerations from previous or existing massive sequencing projects, including Terragenome, The Microbial Earth project, The Gordon and Betty Moore Foundations Virus Sequencing Project, and Meta-HIT.

## Day 1

The 1st International EMP Conference (Twitter hashtag #EMP1, #earthmicrobiome, #earthmicrobiomeproject) was opened by a welcome speech by **Professor Huanming Yang** (Director of BGI) who gave a marvelous introduction to the reason for scientific meetings, which is expounded as ‘to make friends and drive collaboration’. He also reiterated BGI’s excitement at being involved with the EMP, and noted that this study was both ambitious and worthwhile. Professor Yang also Introduced **Professor Rick Stevens** (Argonne National Laboratory, University of Chicago, USA) who gave the keynote for the conference. Professor Stevens discussed the origins, rationale and prospects for the EMP exploring the parallels with the Sloan Foundation’s Digital Sky Survey. He pointed out the EMP’s task was far more difficult, but with much more significant consequences for humankind.

### Session I: Microbial Ecology, the role of the EMP in re-defining research

The first invited speaker was **George Kowalchuck** (The Netherlands Institute of Ecology, The Netherlands) gave an exciting talk about why the EMP is important, and how the generation of comparable data from many ecosystems can help us to redefine our exploration of the microbial world. He argued that it was essential to combine large- and small-scale studies to build up a multidimensional picture of microbial life. Secondly, **Jack A. Gilbert** (Argonne National Laboratory and the University of Chicago, USA) gave a brief welcome and thank you note to the local committee for helping to organize the conference, he then outlined the EMPs fundamental goals, and provided some initial data from the main pilot study of the first 10,000 samples processed. The data were from 5,387 samples and comprised only of 16S rRNA sequences all generated using the same DNA extraction, amplification and sequencing protocol. The samples came from streamwater, soil, marine sediment, human skin, air, coal-beds, lake water, human guts and human oral cavities. The resulting alpha diversity was shown as well as a PCoA plot of all 5,387 samples comprising >210 million sequences of the 16S rRNA gene V4 region generated using Illumina GAIIx amplicon sequencing. Dr. Gilbert also presented results from the Western English Channel study, highlighting several new regional scale models derived from 16S rRNA and metagenomic data generated over a prolonged time series. These models highlighted the end-goal of the EMP, to generate taxonomic and metabolic turnover predictions across space and time. Two offered talks followed. The first was from **Juan Imperial** (Polytechnic University of Madrid, Spain) who made a case for a global survey of legume-rhizobial symbionts. The second was from **Guanghua Wang** (Northeast Institute of Geography and Agroecology, China) who gave the first virus-based talk of the conference and highlighted the need for the EMP to explore viral biodiversity as well, specifically exploring T4-type bacteriophages.

Following a coffee break and group photograph, two further invited talks were given. **Jed Fuhrman** (University of Southern California, USA) gave an excellent overview of the history and taxonomic profiling of marine microbial metagenome and as some of the most studied microbial ecosystems on Earth. He highlighted the absolute necessity for time series studies to determine the variability in any given system. **Janet Jansson** (Lawrence Berkley National Laboratory, USA) provided an exciting overview of the work on the terrestrial microbiome, including the world’s largest metagenomic project, JGI’s Great Prairie Grand Challenge pilot study.

### Session II: Microbial Genomics and Diversity

Following lunch, **Jun Wang** (BGI, China) gave an excellent talk on the genome sequencing of the recent *Escherichia coli* strain from the outbreak in Germany (May/June 2011). He highlighted the importance of the EMP in helping to define the environmental reservoirs of human pathogens. **John Stephen** (Australian Genome Research Facility Ltd, Australia) followed with an introduction to a new initiative to generate a national terrestrial soils map for microbial life in Australia; this was an excellent example of an early stage adopter of the EMP protocols for generating comparable databases of large scale surveys. **Torsten Thomas** (The University of New South Wales, Australia) made an excellent case for exploring the microbial world on physical surfaces, specifically sponges and corals, providing excellent examples of extant data and the lack of comparability among these data. **Janet Seifert** (Rice University, USA) gave a passionate argument for exploring the global diversity of marine stromatolites, which are considered among the oldest microbial ecosystems on Earth, and represent a valuable tool for exploring microbial evolution. **Zhongjun Jia** (Institute of Soil Science, Chinese Academy of Science, China) then provided an excellent example of a country-wide survey of soil microbiota from China, with a focus on the need for collecting detailed and comprehensive environmental data records.

### Session III: EMP case studies

Following the coffee break, **S. Craig Cary** (The University of Waikato, New Zealand) gave an excellent example of a sample collection from Antarctica, for which rich metadata is available, and gave a wonderful example of how to design a regional scale survey. **Tong Zhang** (The University of Hong Kong, Hong Kong) discussed the microbiota of human engineered environments, suggesting the EMP should not overlook these. As an example, he discussed the microbiome of wastewater treatment plants. **Haiyan Chu** (Institute of Soil Science, Chinese Academy of Science, China) gave an excellent example of a global-scale analysis of microbial life in soils, demonstrating that the communities in the arctic were fundamentally similar to communities from many different latitudes.

This closed the official sessions for the first day. The attendees were then offered a tour of the facilities at BGI, Shenzhen, followed by a banquet for all attendees.

## Day 2

### Session IV: Bioinformatic analyses and lessons learned

The first talk on Day 2 was given by **Rob Knight** (University of Colorado at Boulder, USA), who discussed the lessons learned from the Human Microbiome Project and many of the other projects and datasets. **Yangqing Peng** (BGI, China) presented a new suite of bioinformatic tools for exploring genome reassembly from metagenomic data. **Adina Howe** (Michigan State University, USA) also discussed tools for sequence data assembly, including  the need to break-up the data into smaller portions prior to assembly. **Hans-Joachim Ruscheweyh** (Tübingen University, Germany) followed with an excellent presentation of the MEGAN software package for metagenomic data analysis, and how the associated metadata can be used to group metagenomes by environmental parameters.

Following the coffee break, **Yuzhen Ye** (University of Indiana, USA) presented FragGeneScan as a tool for predicting genes in short and error-prone reads. **Tom O. Delmont** (École centrale de Lyon, France) provided some compelling data from the Terragenome Project exploring the soil microbiota from different ecosystems, and the implications of differences in DNA extraction techniques. In a departure from the original agenda, **Hongwei Zhou** (Southern Medical University, China) presented an interesting method for reducing the dominance of abundant members of the community so that a greater proportion of the rare community can be identified. **Scott C. Edmunds** (BGI, China) then presented examples of how to disseminate data following generation, including the idea of data DOIs and citation. **Heshan Lin** (Virginia Tech, USA) discussed the use of graphic processing units (GPUs) for accelerating short-read mapping and local realignment for sequence data. **Cheng-Cang Wu** (Lucigen Corporation, USA) concluded the session with an excellent talk on the use of long-insert clone libraries as another method for exploring the microbial dynamics in different ecosystems.

### Session V:  Data analysis and annotation

Following lunch, **Folker Meyer** (Argonne National Laboratory, University of Chicago, USA) explored the use of cloud computing and MG-RAST to exploit the vast data bonanza being generated by studies similar to the EMP. **Nikos Kyrpides** (DOE-JGI, USA) then discussed the need for comprehensive coverage of genome sequences from cultured isolates to help ground truth observations in metagenomic data, highlighting the Microbial Earth Project (MEP). This project aims to sequence the genomes of all the type strains of Bacteria and Archaea, currently estimated to be around 9,000 taxa. Dr. Kyrpides also suggested that the EMP was so important as to be comparable to the moon race in the 1960s, and, as such, it demanded the need for a government agency (analogous to NASA) in each country to fund and facilitate the effort. This could be realized if the microbiology community would come together and form a distributed research center supporting EMP, which would eventually develop into a Microbial Environmental Genomics Agency (MEGA). **K. Eric Wommack** gave the second viral talk of the conference highlighting the efforts to sequence and survey viral life on Earth. He also introduced Virome as an annotation platform specifically designed for the annotation of viral metagenomic data. **Jack A. Gilbert** then gave a stand-in lecture for **Suzanne Kennedy** (MoBio, USA), which focused on the reproducibility of different DNA extraction methodologies, and introduced some products from MoBio designed at improving the quantity and quality of DNA extracted from different samples.

Following the coffee break, **Lanjuan Li** (Chinese Academy of Engineering, China) gave an excellent talk as the final presenter of the conference, discussing the implications of Hepatitis B infection on human gut microbiota.

To close the meeting, **Jack A. Gilbert** thanked all attendees and speakers, and gave special thanks to **Hanqiao Kang** and **Zimin Zhu** for all their assistance in making the conference such a success.

Following dinner, a panel discussion was held on the need for DNA extraction standards in the EMP. The premise of this working group was to explore the  concerns of the community regarding the adoption of a single DNA extraction methodology for all samples. As already highlighted by two of the talks in the core conference session, DNA extraction can vary from sample to sample, and different methods generate different profiles of the same samples. Importantly, it was evident immediately that no one technique would be an ideal solution. However, it was also made very clear that without a single extraction methodology there could be no absolute comparability between different samples and hence the idea of a systematic global survey would lose much of its value. The protocol adopted by the EMP for the first 10,000 samples pilot study was the MoBio PowerSoil DNA Isolation Kit (both 96-well and single column, depending on number of samples being processed). The manufacturer's protocol was amended with an initial 65 ºC heating step immediately after the addition of the bead solution, and before the shaking step. The outcome of this working group session was that sample and extraction bias will exist no matter which method is adopted, and the need for comparability should override primary concerns that different samples and different taxa will be differentially extracted in different systems. However, some recommendations were made, specifically that, following the initial pilot study, it was imperative that a more comprehensive assessment of the biases associated with different techniques be more thoroughly explored, and that this should be the basis of a second pilot study. Additionally, it was recommended that a robust DNA extraction protocol identified from this pilot study, defined as being able to extract the most DNA from the most environments, be adopted for future EMP studies. Importantly, a review of the DNA extraction protocol would be imperative annually for the continued development and refinement of the EMP. Finally, it was admitted that no one DNA extraction protocol would work for all samples, in that for some samples, the amount of DNA generated would be too low for any analysis. For the pilot study, it was recommended that these samples initially not be included so as to aid the generation of a massive, comparable dataset as quickly as possible. However, as EMP progresses it will be imperative that there be a focus on exploring the level of overlap between different techniques. An archive of these selected EMP sub-samples could be created where different extraction techniques were used that had a known variation to the core extraction protocol.

## Wrap-up

It was agreed that another meeting be held in June 2012 to explore the evolution of the EMP, with the recommendation that the gathering convene in the Netherlands. 
